# “Real-life” data of the efficacy and safety of belantamab mafodotin in relapsed multiple myeloma—the Mayo Clinic experience

**DOI:** 10.1038/s41408-021-00592-3

**Published:** 2021-12-07

**Authors:** I. Vaxman, J. Abeykoon, A. Dispenzieri, S. K. Kumar, F. Buadi, M. Q. Lacy, D. Dingli, Y. Hwa, A. Fonder, M. Hobbs, C. Reeder, T. Sher, S. Hayman, T. Kourelis, R. Warsame, E. Muchtar, N. Leung, R. Go, W. Gonsalves, M. Siddiqui, R. A. Kyle, S. V. Rajkumar, McCullough Kristen, P. Kapoor, M. A. Gertz

**Affiliations:** 1 Division of Hematology, Mayo Clinic, Rochester, MN USA; 2 Institute of Hematology, Davidoff Cancer Center, Rabin Medical Center, Petah- Tikvah, Israel; 3 Sackler Faculty of Medicine Tel-Aviv University, Tel-Aviv, Israel; 4 Division of Hematology, Mayo Clinic, Phoenix, AZ USA; 5 Division of Hematology and Oncology, Mayo Clinic, Jacksonville, FL USA

**Keywords:** Myeloma, Myeloma

## Abstract

Belantamab mafodotin is a highly selective targeted therapy for multiple myeloma. It targets the B cell maturation antigen (BCMA) on plasma cells and showed promising results in several randomized clinical trials. We report the outcomes of 36 patients treated at Mayo Clinic. Our cohort received a median of eight prior lines of therapy. Six patients received belantamab in combination with other medications (pomalidomide, cyclophosphamide, thalidomide), 13 patients (36%) were 70 years or older, two patients had a creatinine of >2.5 mg/dL, and one patient was on dialysis. All three patients with renal failure received full dose belantamab. Chimeric antigen receptor (CAR-T) therapy was used prior to belantamab in seven patients and none of them responded to belantamab therapy. The overall response rate (ORR) was 33% (CR 6%, VGPR 8%, PR 19%), like the ORR reported in the DREAMM-2 trial. Keratopathy developed in 16 patients (43%), grade 1 in six patients, grade 2 in seven patients, and grade 3 in three patients. Eight percent discontinued therapy due to keratopathy. The median PFS and OS was 2 months and 6.5 months, respectively.

## Introduction

Treatment for multiple myeloma (MM) patients that are refractory to proteasome inhibitors (PIs), immunomodulatory agents (IMiDs), and monoclonal antibodies remains unsatisfactory [1–5]. Antibody-drug conjugates (ADCs) are targeted therapies that utilize a monoclonal antibody covalently linked to a highly active cytotoxic payload. The monoclonal antibody component of the ADC selectively targets the cancerous cell and elicits an immune response while delivering the cytotoxic payload directly to the cancerous cell.

Belantamab mafodotin (belamaf® or blenrep®) is the first in class member of the ADCs in MM that utilizes a humanized monoclonal antibody which targets B cell maturation antigen (BCMA) [6–8]. BCMA is a soluble transmembrane glycoprotein that is a member of the tumor necrosis factor superfamily (TNFRSF17) and is overexpressed on mature B cells and plasma cells [8]. The cytotoxic payload is monomethyl auristatin F (MMAF) and it is bound to the antibody via a protease resistant maleimidocaproyl linker [7]. The cytotoxic drug is released only after the ADC has been internalized into the target cell, causing cell death. The drug is administered intravenously at a dose of 2.5 mg/kg every 21 days.

The efficacy of belantamab in relapsed refractory MM (RRMM) patients has been reported in several clinical trials. The phase 1 DREAMM-1 study showed single-agent activity using a dose of 3.4 mg/kg in 35 patients [9]. The overall response rate (ORR) was 60% and the median duration of response (DOR) was 14.3 months. In the subgroup of 13 patients that were refractory to IMiDs and PIs, and exposed to daratumumab, the median progression-free survival (PFS) was 6.2 months, and the ORR was 38.5% [10].

The randomized phase 2 DREAMM-2 study reported the outcomes of 196 RRMM patients that previously received three or more prior lines of therapy (97 at a dose of 2.5 mg/kg and 99 at a dose of 3.4 mg/kg) [11]. The ORR was 30% in the 2.5 mg/kg cohort and 34% in the 3.4 mg/kg cohort. In the recently published 13-month follow-up, the median overall survival (OS) was 13.7 months and the median PFS was 2.8 months [12]. As a result, in August 2020, the drug was approved by the Food and Drug Administration (FDA) as monotherapy for use in RRMM patients that have previously received four or more lines of therapy.

Belantamab is associated with adverse effects typically seen with other myeloma therapies (such as thrombocytopenia, anemia, and infusion-related reactions) [12], but also with a unique adverse effect of ocular toxicity [13, 14]. Grade 3–4 keratopathy (e.g., corneal epithelium changes) was reported in 46% of the DREAMM-2 cohort [12]. Keratopathy led to treatment discontinuation, dose reductions, and dose delays in 4%, 50%, and 95% of the DREAMM-2 cohort, respectively. Baseline and sequential (prior to each dose) ophthalmic examination are mandatory, and prophylactic corticosteroid eye drops are used to mitigate corneal events [15].

The use of belantamab in combination with other agents active in myeloma is currently being prospectively evaluated in the DREAMM-5 [16] and DREAMM-3 study [17]. Results of the phase 1 study that evaluated the combination of belantamab with pomalidomide and dexamethasone (Bela-PD) in 34 RRMM patients were reported at ASH 2020. The treatment scheme was different and included a loading dose and cycles of 28 days. The ORR was 88% and reached 100% among certain subgroups [18].

Clinical trials have restricted patients’ selection, and we report the “real world” efficacy and safety of belantmab in RRMM patients treated at Mayo Clinic.

## Methods

We retrospectively identified all MM patients who received at least one dose of belantamab outside a clinical trial at all three Mayo Clinic sites. The study was approved by the Mayo Clinic Institutional Review Board (IRB). Data were extracted from the medical records and included demographics, baseline disease characteristics, prior therapies, response to belantamab, and adverse events to belantamab therapy.

According to the REMS program, all patients underwent a baseline ophthalmologic evaluation prior to the first dose and serially before every dose. If keratopathy was graded as 2 or more, therapy was held till keratopathy improved to grade1. Therapy was also held for decreased visual acuity. Cooling eye masks were used during belantamab infusion and prophylactic corticosteroid eye drops were used.

The diagnosis and staging of MM were according to consensus criteria [19]. Response and progression were performed according to published criteria. Adverse effects were reported according to CTCAE.

The primary endpoints were ORR, PFS, and OS. PFS was defined as the time from the first day of belantamab administration to progression or death. OS was defined as the time from the first day of belantamab administration to death from any cause. ORR was defined as PR or better. The secondary endpoints included safety. High-risk cytogenetics was defined as t (4;14), t (14; 16), t (14;20), del 17p, and 1q amp.

Categorical variables were described by numbers and percentages, and the difference between groups was evaluated using the chi-square test (for normally distributed variables) or by the Fischer exact test (for non-normally distributed variables). Continuous variables were described by mean and standard deviation and compared using a student *T*-test (for normally distributed parameters), or by medians and interquartile range (IQR) and compared by the Wilcoxon signed-rank tests (for non-normally distributed variables).

Kaplan–Meier method was used for PFS, and OS analysis and all statistical tests were two-sided and *P*-values of <0.05 were significant. Statistical analysis was carried out using JMP 14 (SAS Institute, Cary, NC) statistical software.

## Results

Between September 2020 and June 2021, 36 patients received at least one dose of belantamab at Mayo Clinic (25 at Mayo Rochester, 6 at Mayo Arizona, and 5 at Mayo Florida). Thirty (83%) received belantamab monotherapy and 6 patients (17%) received belantamab in combination with pomalidomide (*n* = 3), cyclophosphamide (*n* = 2) and thalidomide (*n* = 1).

### Baseline characteristics

Table 1 shows the baseline characteristics of all patients at diagnosis. The median age at myeloma diagnosis was 61 years (range 37–83) and 23 patients (64%) were men. The median age at belantamab administration was 67 years (IQR 59–74) and 13 patients (36%) were 70 years or older. 14 of 34 patients (41%) had high-risk cytogenetics at diagnosis. One patient was black. The median creatinine at the time of the first belantamab dose was 1.09 mg/dL (IQR 0.89–1.48). Two patients had a creatinine of 2.5 and one patient was on dialysis, all three received full dose belantamab (2.5 mg/kg).

**Table 1:** Baseline characteristics at diagnosis of study population.

Variable	Cohort *N* = 36
Age, mean years (range)	61 (37–83)
Male, *n* (%)	23 (64)
Time from Dx to belanatamb, years (IQR)	7 (4–11)
Median Plasma cells at diagnosis, % (IQR)	70% (40–80)
Median hemoglobin, g/dL (IQR)	9.9 (8.3–12.3)
Median creatinine, mg/dL (IQR)	1.1 (0.9–2.3)
Median calcium, mg/dL (IQR)	9.8 (9.1–12.3)
Median beta 2 microglobulin, IQR	3.9 (2.5–9.5)
Median LDH, U/L (IQR)	173 (137–208)
Median albumin, g/dL (IQR)	3.6 (3.4–4)
Median lines of prior therapies, *n* (IQR)	8 (7–11)
ISS, n (%)	
1	9 (25)
2	6 (17)
3	12 (33)
Missing	9
High risk genetics at diagnosis, *n* (%)	14 (41)
Extramedullary disease, *n* (%)	5 (14)
Plasma cell leukemia, *n* (%)	3 (8)
ASCT, *n* (%)	27 (75)
Bortezomib refractory, *n* (%)	36 (100)
Lenalidomide refractory, *n* (%)	36 (100)
Pomalidomide refractory, *n* (%)	36 (100)
Carfilzomib refractory, *n* (%)	36 (100)
Daratumumab refractory, *n* (%)	36 (100)
Median PLT at belantamab initiation (IQR)/mcL	69 (43–106)
Number of patients treated with PLT < 75/mcL (%)	19 (53)
Median ANC at belantamab initiation (IQR)/ mcL	2.5 (1.48–3.33)
Number of patients treated with ANC < 750/mcL, (%)	1 (3%)

*N* number, *PI* proteasome inhibitors, *IMiD* immunomodulatory drugs, *ISS* international staging system, *ASCT* autologous stem cell transplantation, *PLT* platelets, *ANC* absolute neutrophil count.

Twenty-seven patients (75%) underwent prior autologous stem cell transplantation (ASCT), two patients underwent a second ASCT. All patients were refractory to daratumumab, PIs, and IMiDs. Seven patients (19%) had received prior Chimeric antigen receptor (CAR-T) therapy.

### Treatment and response

The median time from diagnosis to belantamab first dose administration was 7 years (IQR 4–11) and the median lines of therapy prior to belantamab was 8 (IQR 7–11). The median number of belantamab doses received was 3 (range 1–6). The overall response (ORR) rate to belantamab was 33%. Two patients (6%) achieved complete response (CR), three patients (8%) achieved very good partial response (VGPR), and seven patients (19%) achieved partial response (PR). Ten patients (28%) achieved stable disease (SD) and 13 patients (36%) progressed while on belantamab therapy (data on disease status prior to belantamab therapy was not evaluated in one patient so we were unable to assess the depth of his response). The median duration of response was 5 months (range 2–11).

Five patients (14%) are still on therapy. Of these patients two achieved CR, one VGPR and one achieved PR (one was unevaluable for response) and the median duration of therapy of these five patients that are still receiving belantamab therapy is 7 months (range 3–11 months). Reasons for treatment discontinuation were PD in 28 patients (77%) and keratopathy in three patients (8%). None of the patients that were treated with CAR-T before belantamab responded to therapy (2 SD, 5 PD).

### Survival outcomes

At data cutoff 18 patients (50%) are alive. Eighteen patients died of MM complications. The median follow-up for the entire cohort of surviving patients was 6 months (IQR 3.5–7).

The median PFS and OS for the whole cohort were 2 months (95% CI 1–3) and 6.5 months (95% CI 3-NR), respectively. Two patients died within the first cycle of belantamab (due to PD), and overall, 12 died within the first 100 days of belantamab therapy. All the patients that died in our cohort (18 patients), died due to progressive disease.

### Adverse effects

Twelve patients were hospitalized during belantamab therapy (33%). The reasons for hospitalization were related to myeloma in four patients (one patient with malignant ascites, one with hypercalcemia, two patients for pain management). Three patients were hospitalized due to thrombocytopenia (related to the myeloma), and one due to an infusion related reaction (IRR). Other reasons for hospitalization were infection (soft tissue abscess, *n* = 1), proctitis (*n* = 1), colitis (*n* = 1) and tumor lysis (*n* = 1). Keratopathy developed in 16 patients (44%). Keratopathy was grade 1 in six patients, grade 2 in seven patients, and grade 3 in three patients. Only one patient had corneal erosions and six patients reported decreased visual acuity, (four had grade 2 keratopathy, one had grade 1 keratopathy, and one grade 3). Two patients had mild infusion related reactions (IRR). Two patients reported gaining 3.5 kg after therapy with belantamab was discontinued.

## Discussion

Our cohort of 36 patients treated with belantamab for RRMM is a heavily pretreated group of patients with a median of eight prior lines of therapy and a median time from diagnosis to first belantamab dose of 7 years. Six patients (17%) in our cohort received belantamab in combination with other agents (pomalidomide, cyclophosphamide and thalidomide) and seven patients (19%) received another anti BCMA agent (CAR-T) prior to belantamab therapy. We found a median PFS of 2 months, a median OS of 6.5 months and the ORR was 33%. Our patients were treated based on need without the constraints seen in a clinical trial. Three of our patients had creatinine >2.5 mg/dL excluded from the DREAMM trials, 19 patients (53%) had a platelet count <75,000/mcL and one patient had neutrophils less than 750/mcL, which would not have been enrolled in most trials of RRMM and reflects the true needs of this population.

Our results are in line with the prospective trials that evaluated belantamab in refractory MM patients. In the DREAMM-1 [9, 10], the response rate was over 70% for patients not previously exposed to daratumumab. However, the subgroup of 13 patients that were refractory to IMiDs and PIs, and exposed to daratumumab, which is like our cohort, had an ORR of 38.5%. Moreover, the median PFS of 6.2 months in this subgroup was significantly shorter than the entire cohort. In the DREAMM-2 trial, 100% of the patients were triple refractory, as in our cohort. The ORR in the DREAMM-2 was 31% and the mPFS 2.8 months [12]. Table 2 compares our cohort to the DREAMM-2 cohort 2.5 mg/kg in terms of patient characteristics and outcomes. In our cohort, only one patient received belantamab after only four lines of therapy versus 16 patients (16%) that received belantamab after ≤4 lines of therapy in the DREAMM-2 trial. Our cohort included three patients that had renal failure that would make them ineligible for the DREAMM-2 trial and seven patients that underwent CAR-T therapy prior to belantamab therapy. This may explain our shorter OS (6.5 months) versus the 13.8 months OS reported in the DREAMM-2 study. Deeper reponses were seen in the DREAMM-2 vs. our cohort (Table 2). In the DREAMM-2 trial, the response deepened over time, so the difference in depth of response may be attributed to our shorter follow up (the median follow-up in our cohort was 6 months and the median follow-up in the DREAMM-2 was 13 months).

**Table 2:** comparison of our cohort to the DREAMM-2 cohort.

Variable	Our cohort (*N* = 36)	DREAMM- 2 cohort 2.5 mg/kg (*N* = 97)
Median number of prior lines	8	7
The median time from diagnosis to first belantamab dose (years)	7	5.5
Median age at belantamab administration (years)	67	65
Extramedullary disease (%)	14	23
GFR < 30 (%)	8	2
Elderly	13 (36%) over 70	13 (13%) over 75
HR cytogenetics, *n* (%)	14 (41%)	41 (42%)
Prior CAR-T therapy, *n* (%)	7 (19%)	0
Median PFS (month)	2	2.8
Median OS (months)	6.5	13.8
ORR (%)	33	32
CR/sCR	6	7
VGPR	8	11
PR	19	13
Keratopathy	43% (G1 = 6 pt, G2 = 7 pt, G3 = 3 pt, G4 or 5 = 0)	67% (G1–2 = 41 pt, G3 = 26 pt, G4 or 5 = 0)
IRR	2 (5%)	20 (21%)

*CR* complete response, *VGPR* very good partial response, *PR* partial response, *IRR* infusion related reaction, *GFR* glomerular filtration rate, *HR* high risk.

In recent years, three BCMA targeted therapies demonstrating high efficacy have been evaluated in RRMM: ADCs (belantamab), CAR-T [5, 20], and bispecific T cell engagers [21, 22]. The results of the CAR-T trials showed an ORR of over 80% in a heavily pretreated patient population. However, relapses are observed in patients treated with CAR-T, with a 12 months PFS of 77% in the CARTITUDE-1 [20] and a median PFS of 8.8 months in the KarMMa trial [5]. The mechanisms of resistance to anti-BCMA include BCMA antigen shedding [23, 24], anti-drug antibodies [25], antigen escape [26, 27], the emergence of an immunosuppressive microenvironment, and T-cell exhaustion [28]. Currently, most published trials excluded patients with prior BCMA-directed therapy exposure. However, retreatment with an anti BCMA therapy is currently being investigated. There are case reports of sequential use of these agents [29, 30] all demonstrating clinical activity of BCMA-targeted therapies in patients previously treated with other BCMA-targeted agents. The long-term response durability of all anti-BCMA agents remains unknown currently and the appropriate sequence of these therapies is unclear. In our cohort, seven patients were treated with CAR-T prior to belantamab therapy. None of these patients responded to belantamab therapy.

Six patients received belantamab in combinations (pomalidomide (*n* = 3), cyclophosphamide (*n* = 2), and thalidomide (*n* = 1). Treating MM with triplets had been shown to improve outcomes, and combining belantamab with pomalidomide showed high response rates [18]. Several phase 3 clinical trials are currently investigating belantamab in combination with novel agents: pomalidomide (NCT04162210), bortezomib (NCT 04246047) and lenalidomide (NCT 04061126). No safety issues were seen in patients treated with belantamab in combinations in our cohort. All patients started with one month of belantamab monotherapy and the second agent was added in the second cycle.

Toxicities that were seen in our cohort were previously reported in the DREAMM trials. The hospitalization rate was 33% and were mostly related to MM complications (malignant ascites, hypercalcemia, pain management). Infections were documented in two patients. The only previously unreported toxicity was seen in one patient hospitalized due to a suspected TLS. TLS was not reported in either DREAMM trials. Adverse events of special interest include corneal events, thrombocytopenia, and infusion related reactions (IRR). IRR was seen in only two patients in our cohort, both grade 3, and causing one hospitalization. These are lower rates than reported in clinical trials (all grade 35%) and this may have been caused by underreporting of grade 1–2 IRR. In the DREAMM-2 trial the grade 3 IRR was 3%. Three patients were hospitalized due to thrombocytopenia, all related to the myeloma. Keratopathy was grade 1 in six patients, grade 2 in seven patients, and grade 3 in three patients. Only one patient had corneal erosions and six patients reported decreased visual acuity, (four had grade 2 keratopathy, one had grade 1 keratopathy, and one grade 3). While some clinicians argue that this drug is simply too toxic, we argue that monitoring ocular toxicity by slit lamp rather than visual acuity symptoms, results in therapy delays which may not be clinically relevant. This is even more important since this is a very refractory patient population that have limited therapy options if dose delays result in relapse.

Our study has several limitations. First, this is a retrospective study. Second, it includes only 36 patients so we cannot make inferences about patients that receive belantamab in combinations, or in renal failure. However, to the best of our knowledge this is the largest “real-life” cohort of RRMM patients treated with belantamab.

In conclusion, the results of “real life” belantamab are similar to those published in the RCTs, and despite some promise, the role of belantamab in the era of other BCMA directed therapy (CAR-T, bispecific T cell engagers) remains unclear.

## References

[1] Gandhi UH, Cornell RF, Lakshman A, Gahvari ZJ, McGehee E, Jagosky MH (2019). Outcomes of patients with multiple myeloma refractory to CD38-targeted monoclonal antibody therapy. Leukemia.

[2] Grosicki S, Simonova M, Spicka I, Pour L, Kriachok I, Gavriatopoulou M (2020). Once-per-week selinexor, bortezomib, and dexamethasone versus twice-per-week bortezomib and dexamethasone in patients with multiple myeloma (BOSTON): a randomised, open-label, phase 3 trial. Lancet.

[3] Attal M, Richardson PG, Rajkumar SV, San-Miguel J, Beksac M, Spicka I (2019). Isatuximab plus pomalidomide and low-dose dexamethasone versus pomalidomide and low-dose dexamethasone in patients with relapsed and refractory multiple myeloma (ICARIA-MM): a randomised, multicentre, open-label, phase 3 study. Lancet.

[4] Dimopoulos M, Quach H, Mateos MV, Landgren O, Leleu X, Siegel D (2020). Carfilzomib, dexamethasone, and daratumumab versus carfilzomib and dexamethasone for patients with relapsed or refractory multiple myeloma (CANDOR): results from a randomised, multicentre, open-label, phase 3 study. Lancet.

[5] Munshi NC, Anderson LD, Shah N, Madduri D, Berdeja J, Lonial S (2021). Idecabtagene vicleucel in relapsed and refractory multiple myeloma. N Engl J Med.

[6] Lee L, Bounds D, Paterson J, Herledan G, Sully K, Seestaller-Wehr LM (2016). Evaluation of B cell maturation antigen as a target for antibody drug conjugate mediated cytotoxicity in multiple myeloma. Br J Haematol.

[7] Tai YT, Mayes PA, Acharya C, Zhong MY, Cea M, Cagnetta A (2014). Novel anti-B-cell maturation antigen antibody-drug conjugate (GSK2857916) selectively induces killing of multiple myeloma. Blood.

[8] Montes de Oca R, Alavi AS, Vitali N, Bhattacharya S, Blackwell C, Patel K (2021). Belantamab mafodotin (GSK2857916) drives immunogenic cell death and immune-mediated anti-tumor responses in vivo. Mol Cancer Ther.

[9] Trudel S, Lendvai N, Popat R, Voorhees PM, Reeves B, Libby EN (2018). Targeting B-cell maturation antigen with GSK2857916 antibody-drug conjugate in relapsed or refractory multiple myeloma (BMA117159): a dose escalation and expansion phase 1 trial. Lancet Oncol.

[10] Trudel S, Lendvai N, Popat R, Voorhees PM, Reeves B, Libby EN (2019). Antibody-drug conjugate, GSK2857916, in relapsed/refractory multiple myeloma: an update on safety and efficacy from dose expansion phase I study. Blood Cancer J.

[11] Lonial S, Lee HC, Badros A, Trudel S, Nooka AK, Chari A (2020). Belantamab mafodotin for relapsed or refractory multiple myeloma (DREAMM-2): a two-arm, randomised, open-label, phase 2 study. Lancet Oncol.

[12] Lonial S, Lee HC, Badros A, Trudel S, Nooka AK, Chari A (2021). Longer term outcomes with single-agent belantamab mafodotin in patients with relapsed or refractory multiple myeloma: 13-month follow-up from the pivotal DREAMM-2 study. Cancer.

[13] Bausell RB, Soleimani A, Vinnett A, Baroni MD, Staub SA, Binion K (2021). Corneal changes after belantamab mafodotin in multiple myeloma patients. Eye Contact Lens.

[14] Wahab A, Rafae A, Mushtaq K, Masood A, Ehsan H, Khakwani M (2021). Ocular toxicity of belantamab mafodotin, an oncological perspective of management in relapsed and refractory multiple myeloma. Front Oncol.

[15] Lonial S, Nooka AK, Thulasi P, Badros AZ, Jeng BH, Callander NS (2021). Management of belantamab mafodotin-associated corneal events in patients with relapsed or refractory multiple myeloma (RRMM). Blood Cancer J.

[16] Nooka AK, Weisel K, van de Donk NW, Routledge D, Otero PR, Song K (2021). Belantamab mafodotin in combination with novel agents in relapsed/refractory multiple myeloma: DREAMM-5 study design. Future Oncol.

[17] Weisel K, Hopkins TG, Fecteau D, Bao W, Quigley C, Jewell RC, et al. editor. Dreamm-3: a phase 3, open-label, randomized study to evaluate the efficacy and safety of belantamab mafodotin (GSK2857916) monotherapy compared with pomalidomide plus low-dose dexamethasone (Pom/Dex) in participants with relapsed/refractory multiple myeloma (RRMM). Blood. 2019;134:1900.

[18] Suzanne Trudel M, McCurdy A, Sutherland HJ, Louzada ML, Venner CP, White DJ, et al. editor. Part 1 results of a dose finding study of belantamab mafodotin (GSK2857916) in combination with pomalidomide (POM) and dexamethasone (DEX) for the treatment of relapsed/refractory multiple myeloma (RRMM). Blood. 2020;136:abstract725.

[19] Rajkumar SV, Dimopoulos MA, Palumbo A, Blade J, Merlini G, Mateos MV (2014). International Myeloma Working Group updated criteria for the diagnosis of multiple myeloma. Lancet Oncol.

[20] Berdeja JG, Madduri D, Usmani SZ, Jakubowiak A, Agha M, Cohen AD (2021). Ciltacabtagene autoleucel, a B-cell maturation antigen-directed chimeric antigen receptor T-cell therapy in patients with relapsed or refractory multiple myeloma (CARTITUDE-1): a phase 1b/2 open-label study. Lancet.

[21] Topp MS, Duell J, Zugmaier G, Attal M, Moreau P, Langer C (2020). Anti-B-cell maturation antigen BiTE molecule AMG 420 induces responses in multiple myeloma. J Clin Oncol.

[22] Usmani SZ, Mateos MV, Nahi H, Krishnan AY, Niels WCJ, van de Donk, et al. editors. Phase I study of teclistamab, a humanized B-cell maturation antigen (BCMA) x CD3 bispecific antibody, in relapsed/refractory multiple myeloma (R/R MM). ASCO. 2020;38:15.

[23] Laurent SA, Hoffmann FS, Kuhn PH, Cheng Q, Chu Y, Schmidt-Supprian M (2015). γ-Secretase directly sheds the survival receptor BCMA from plasma cells. Nat Commun.

[24] Chen H, Li M, Xu N, Ng N, Sanchez E, Soof CM (2019). Serum B-cell maturation antigen (BCMA) reduces binding of anti-BCMA antibody to multiple myeloma cells. Leuk Res.

[25] Xu J, Chen LJ, Yang SS, Sun Y, Wu W, Liu YF (2019). Exploratory trial of a biepitopic CAR T-targeting B cell maturation antigen in relapsed/refractory multiple myeloma. Proc Natl Acad Sci USA.

[26] Da Vià MC, Dietrich O, Truger M, Arampatzi P, Duell J, Heidemeier A (2021). Homozygous BCMA gene deletion in response to anti-BCMA CAR T cells in a patient with multiple myeloma. Nat Med.

[27] Samur MK, Fulciniti M, Aktas Samur A, Bazarbachi AH, Tai YT, Prabhala R (2021). Biallelic loss of BCMA as a resistance mechanism to CAR T cell therapy in a patient with multiple myeloma. Nat Commun.

[28] Leblay N, Maity R, Barakat E, McCulloch S, Duggan P, Jimenez-Zepeda V (2020). editors Cite-seq profiling of T cells in multiple myeloma patients undergoing BCMA targeting CAR-T or bites immunotherapy. Blood.

[29] Gazeau N, Beauvais D, Yakoub-Agha I, Mitra S, Campbell TB, Facon T (2021). Effective anti-BCMA retreatment in multiple myeloma. Blood Adv.

[30] Cohen AD, Garfall AL, Dogan A, Lacey SF, Martin C, Lendvai N (2019). Serial treatment of relapsed/refractory multiple myeloma with different BCMA-targeting therapies. Blood Adv.

